# Supplementary figures and images for: Comparative Genomics Used to Predict Virulence Factors and Metabolic Genes among Monilinia Species
Source: J Fungi (Basel). 2021 Jun 8;7(6):464. doi: 10.3390/jof7060464 (PMC8228255; doi:10.3390/jof7060464)

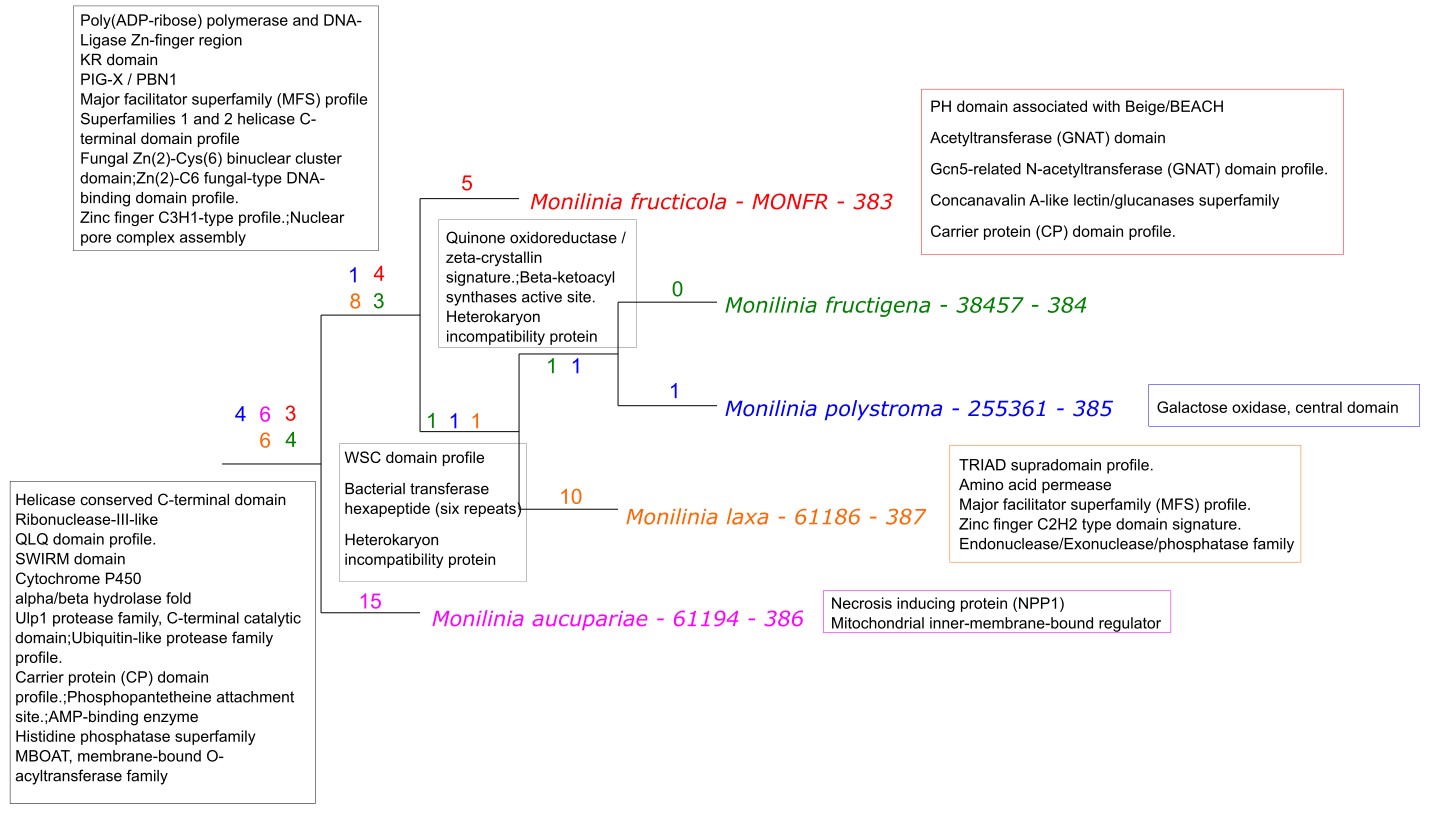

Supplement: Supplementary file 1 [file jof-07-00464-s001.zip › Figure S1.jpg]

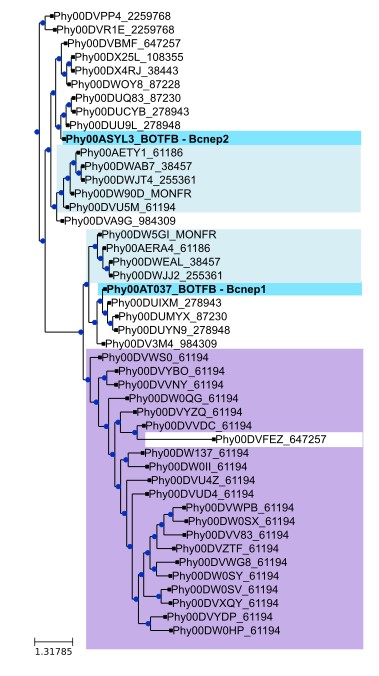

Supplement: Supplementary file 1 [file jof-07-00464-s001.zip › Figure S2.jpg]

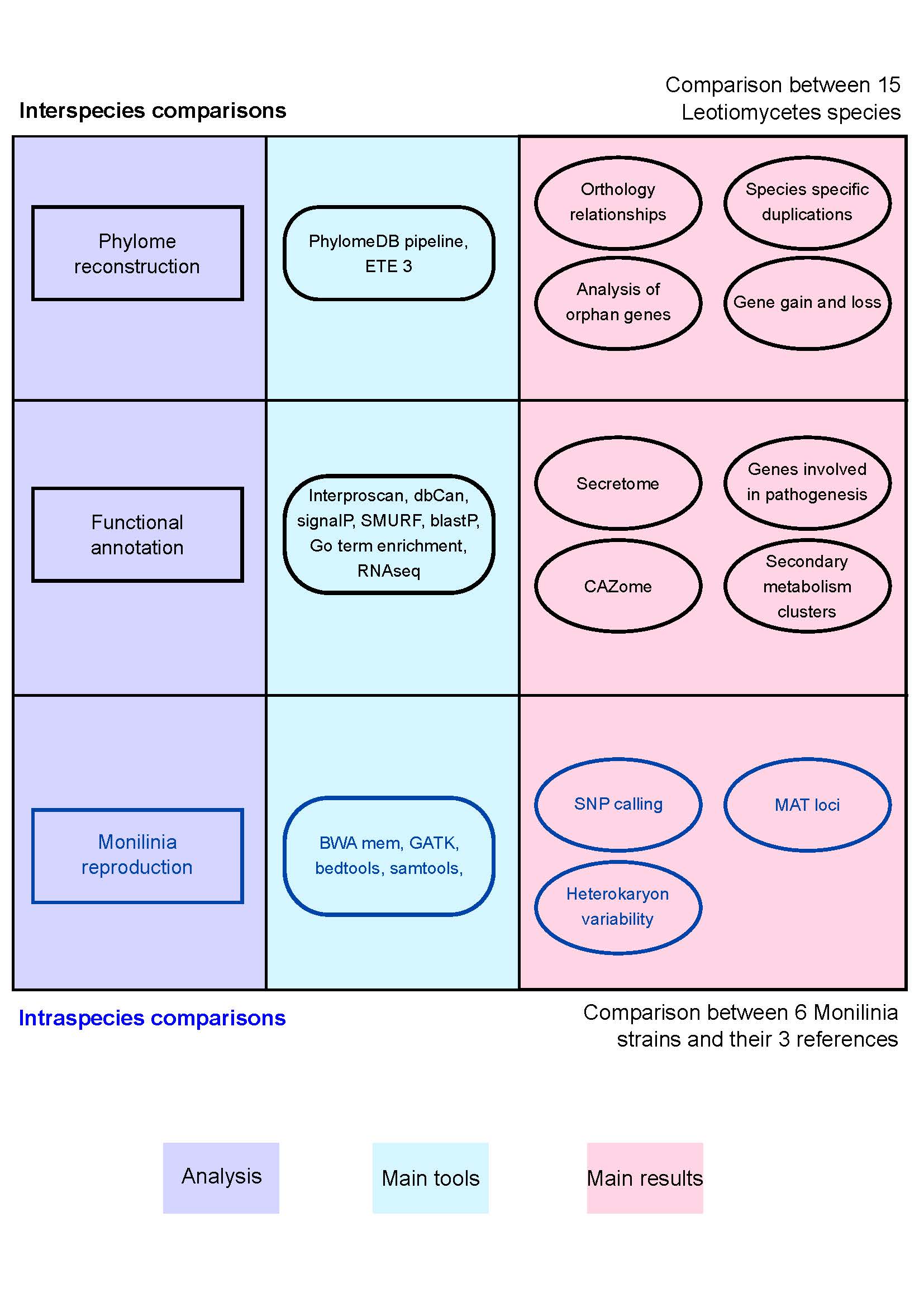

Supplement: Supplementary file 1 [file jof-07-00464-s001.zip › Figure S3.jpg]

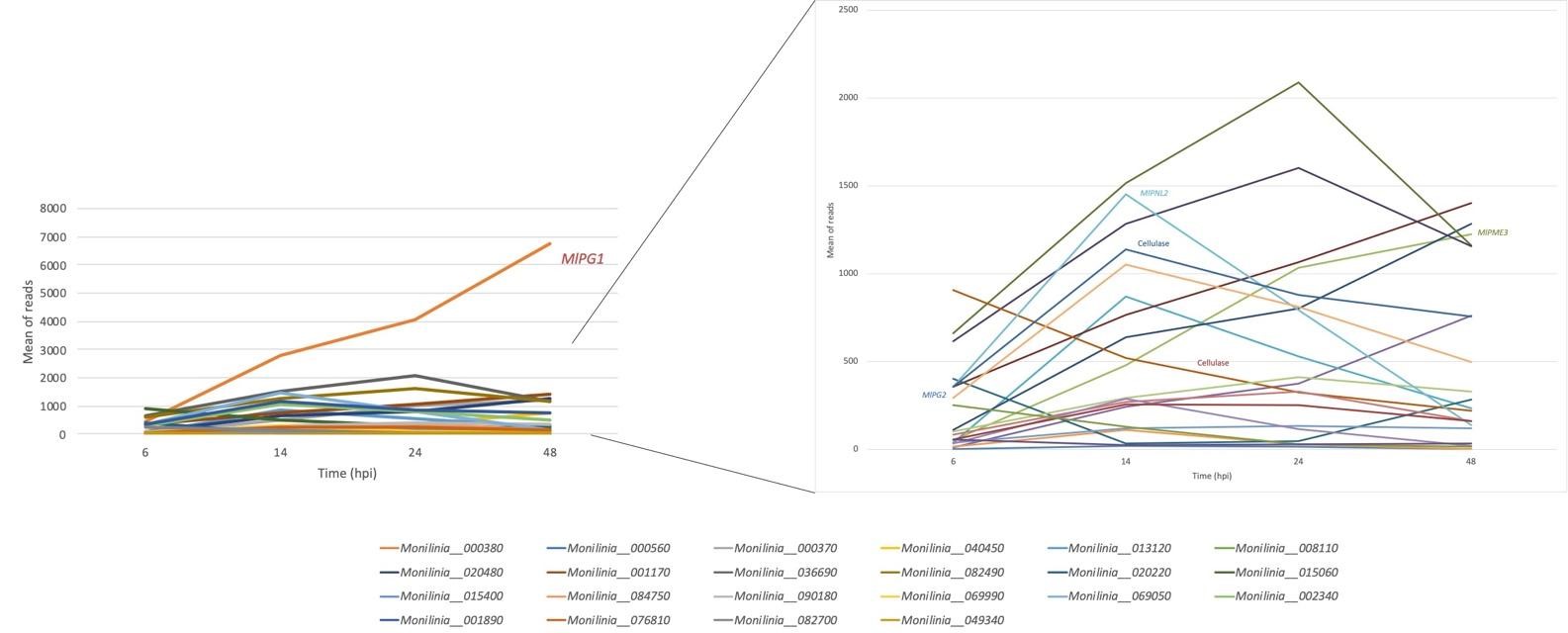

Supplement: Supplementary file 1 [file jof-07-00464-s001.zip › Figure S4.jpg]

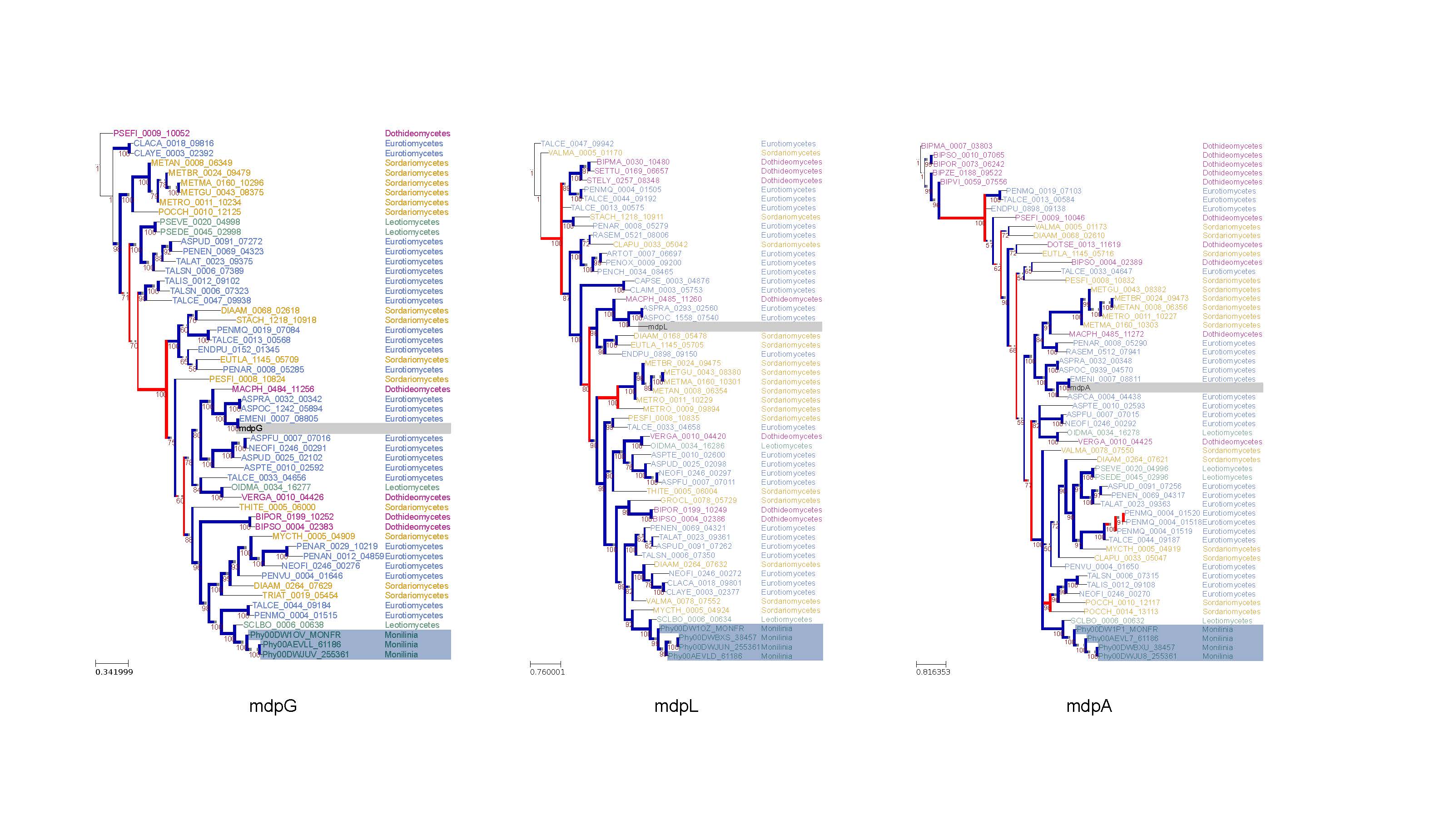

Supplement: Supplementary file 1 [file jof-07-00464-s001.zip › Figure S5_revised.jpg]
